# Working together in clinical pathology: An interprofessional education initiative for dentistry, oral health, and medical laboratory science teachers and students

**DOI:** 10.15694/mep.2020.000009.1

**Published:** 2020-01-13

**Authors:** Hanna Olson, Catherine Ronayne, Megan Anakin, Alison Meldrum, Alison Rich

**Affiliations:** 1 University of Otago

**Keywords:** dentistry, interprofessional education, medical laboratory science, oral health, orofacial pathology, patient-centred care

## Abstract

This article was migrated. The article was not marked as recommended.

**Introduction**. In the health professional education literature, there is a need for information about the teaching and learning of medical laboratory sciences for clinical practice. The goal of this reflection-on-practice is to describe how an orofacial pathology interprofessional education (IPE) initiative was designed and implemented.

**Innovation**. The designers of this initiative were teachers from dentistry, oral health, and medical laboratory science. The designers used six interprofessional competences (patient-centred care, role clarification, team functioning, collaborative leadership, communication, and cultural practice) to guide their construction of teaching and learning resources. The initiative required students to work collaboratively with a given patient case to develop a differential diagnosis, prepare a treatment plan, present their case to classmates and staff members, and describe how they worked together to address the orofacial pathology in their case.

**Evaluation**. The designers collected and considered evaluation information including the learning resources used, logistical arrangements for the initiative, and student ratings about interprofessional competencies.

**Outcomes**. In general, the designers felt that they had successfully designed and implemented an IPE initiative that met five of the six interprofessional competencies. The designers did not feel that the cultural practice competency was adequately addressed in this version of the initiative.

**What next?** Given the acceptability of this initiative to the designers, facilitators, and students, the next step is to consider the feasibility of scaling-up this small voluntary IPE initiative into a permanent component of the dentistry, oral health, and medical laboratory science programmes. Aspects to consider include staffing, scheduling, assessment, and cultural perspectives.

## Introduction

This article reports a reflection-on-practice and describes how an interprofessional education (IPE) initiative involving dental, oral health, and medical laboratory science students was designed and implemented. This initiative originated to address the need to provide IPE to undergraduate students preparing for professions in dentistry and the medical laboratory sciences at the University of Otago in Dunedin, New Zealand. Ethical approval was not sought from the Human Ethics Committee at the University of Otago for this descriptive report. A research study is planned to evaluate the next phase of this initiative. This report addresses a gap in the health professional education literature about the teaching and learning of medical laboratory sciences for clinical practice.

An increasingly important aspect of healthcare involves collaboration of interprofessional teams to provide patient care. To help develop a collaborative workforce, healthcare educators established interprofessional education (IPE) an effective method of building a team approach to healthcare ( Davis *et al.*, 1995). The term IPE is commonly used to refer to opportunities where two or more professions learn together to develop knowledge, skills, and attitudes for collaborative practice and with the goal of improving patient care. IPE activities tend to involve practitioners in fields such as medicine, physiotherapy, pharmacy, nursing and occupational therapy to a greater extent than dentistry and medical laboratory science; and this situation is also true for undergraduate IPE activities ( Brown and Miller, 2016). Consequently, there is a need to report IPE activities involving undergraduate students preparing for professions in dentistry and the medical laboratory sciences.

## Innovation

One factor related to the success of IPE activity can be the selection of a topic that presents authentic opportunities for healthcare professionals to interact as they provide patient care. When students from multiple professions are exposed to complex situations of patient care, it allows them to establish connections with other professions and enhance the understanding of the roles and responsibilities of individuals within interprofessional teams ( Thompson *et al.*, 2016). Dentists and oral health therapists are often the first point of contact when orofacial pathology is suspected. Having knowledge of oral pathology, dental clinicians can become involved in diagnosing pathology such as the precursor lesions of oral carcinoma ( Subramanyam, 2014). Oral health therapists may refer patients to dentists, who may undertake procedures such as a biopsy, or may order diagnostic tests which would be processed by medical laboratory scientists. Medical laboratory scientists tend to work behind the scenes, this IPE activity was intended to reinforce the critical nature of their role within the healthcare team ( Dolce, Parker and Werrlein, 2012). Additionally, students in medical laboratory science programmes will gain valuable experience dealing with multiple patients, phone calls, and difficulties in interprofessional communication ( Brown and Miller, 2016). Therefore, the topic of orofacial pathology was selected for the focus of a pilot IPE initiative.

The designers of this initiative were all but the third author of this article. The initiative was specifically designed for students in their second year of the Bachelor of Oral Health, third year of the Bachelor of Medical Laboratory Science and fourth year of the Bachelor of Dental Surgery programmes at the University of Otago. To emphasize interprofessional competencies ( Canadian Interprofessional Health Collaborative, 2010), the initiative was designed so students would use their current knowledge rather than learn new scientific facts. The dental and oral health students already had experienced the provision of patient-centred care. Both groups of students had knowledge of orofacial pathology from previous coursework. For medical laboratory science students, the initiative provided a preview of potential clinical interactions before they began their 30-week clinical placement. Therefore, the overarching learning goal for the orofacial pathology IPE initiative was for students to discuss patient-centred care related to their knowledge to diagnose, investigate oral diseases and their implications to the oral environment in a collaborative, respectful, and responsible way.

The initiative used a case-study approach. Three cases were written collaboratively by academics from dentistry, including a specialist oral pathologist, oral health and medical laboratory science. The cases were based on their experiences with patients in their clinical practice and included presentations of oral manifestations of acute leukaemia, drug-associated gingival bleeding, and an ulcer subsequently diagnosed as oral cancer. Each case included patient details such as age, current concerns, general/physical appraisal, social, medical and dental history, tobacco smoking history, and clinical findings from a recent dental visit. In addition to clinical notes, each case included at least one clinical photo, histological image, and/or laboratory results from recent blood tests. (See Appendix 1 for details of Cases 1, 2, and 3).

The initiative was conducted over four weeks. It was supported by three one-hour interactive sessions at weeks 1, 3 and 4. The initiative had three facilitators, one from each participating programme. The first six students from each programme to respond to an invitation to participate were included in the project. Participating students were organised into groups of six with two students from each programme. Students were encouraged to communicate and collaborate between sessions. To avoid timetable clashes, meetings were held between scheduled classes with lunch provided. The student workload was approximately ten hours.

In the first session, facilitators gave a brief overview of IPE and the learning objectives of the project. Students introduced themselves to one another and discussed their professional roles in their groups. Students were encouraged to discuss the role and responsibility of each profession included referral pathways to foster holistic, patient-centred care. In the second session, students worked in their groups with facilitator support to develop a holistic care plan for their patient case. The third session was held in a small lecture theatre with an audience of interested students and staff. Each group presented a treatment plan that included a description of each profession’s roles in the process of managing their oral pathology case. After the third session, students were issued an electronic certificate acknowledging their participation and effort in the initiative.

## Evaluation

In addition to reviewing the teaching and learning resources and logistical arrangements required for this initiative, the designers of the initiative constructed a student questionnaire to collect information to evaluate the feasibility of incorporating the initiative permanently into the undergraduate dental, oral health, and medical laboratory science programmes. Items in the questionnaire reflected six interprofessional competencies. Five competency domains were from *the National Interprofessional Competency Framework* ( Canadian Interprofessional Health Collaborative, 2010). The sixth competency was ‘cultural practice’ to reflect the importance of demonstrating culturally appropriate professional behaviours in the New Zealand context ( New Zealand Ministry of Health, 2003). The resulting questionnaire asked students to rate nine statements using a five-point Likert-type scale (‘agree to a very small extent’ = 1 and ‘agree to a very large extent’ = 5) and to provide written comments about the initiative (See Table 1).

**Table 1.** Evaluation Questionnaire Organised by Correspondence to Five Interprofessional Competencies ( Canadian Interprofessional Health Collaborative, 2010)

Questionnaire Statement	Interprofessional Competency
Patients would ultimately benefit if health professionals work together to solve patient problems.	
	**Patient-Centred Care**
Interprofessional learning (IPL) has increased my ability to work collaboratively to provide patient-centred care.	
Interprofessional learning helped me articulate my role and role boundaries in a health and social care team.	
	**Role Clarification**
Through IPL I have become more knowledgeable about the roles of a range of health professionals.	
IPL helped me to think about how a variety of different health professionals can work effectively in a team.	
	**Team Functioning**
As a result of IPL, I have a better understanding of roles, activities and skills of different professions.	
IPL showed me that leadership is fluid and can change among team members according to patient-need.	**Collaborative Leadership**
My skills in communicating with other health professionals improved through learning with students from varied health care professions.	**Interprofessional Communication**
Interprofessional learning helped me to model from students to develop and use skills for culturally sensitive communication.	**Cultural Practice**

As part of regular teaching practice at the University of Otago, evaluation information was collected electronically, voluntarily with informed consent, and anonymously from students after the third interactive session. Evaluations were managed by the University’s Quality Advancement Unit. The designers of the initiative reviewed and summarised evaluation information.

## Outcomes

In general, the designers felt that they had successfully designed and implemented an IPE initiative involving dental, oral health, and medical laboratory science students. The designers considered that the teaching and learning resources were fit for purpose and that the logistical arrangements were acceptable for a small scale pilot involving 3 facilitators and 18 students. The designers noted that students rated items corresponding to five of the six interprofessional competencies highly. Students’ written comments were interpreted to suggest that the initiative addressed the competencies of patient-centred care, role clarification, team functioning, collaborative leadership, and communication effectively. Of concern to the designers was the moderate agreement from students that the initiative addressed the cultural practice competency and that there were no written comments provided by students about this aspect of the initiative.

## What’s Next?

Given the successful design and implementation of this orofacial pathology IPE initiative, the designers are planning to expand and research this initiative in the future. The evaluation information interpreted by the designers to suggest that when students from three health professional programmes interacted with one another to manage a case in orofacial pathology, they appreciated learning with, from, and about each other. This finding is consonant with evaluation results reported previously, where 80% of students from different health professional programmes agreed that a course in interprofessional learning would have an effect on their future relationships with other professional groups ( Tsakitzidis *et al.*, 2015). A notable design issue to be addressed is the cultural practice competency. Given the importance of developing culturally appropriate professional behaviours in New Zealand ( New Zealand Ministry of Health, 2003) this feature will be enhanced before the initiative is repeated in the future.

When considering the usefulness of applying ideas from this reflection-on-practice for use elsewhere, it is important to note that only a small number of facilitators and students were involved in piloting this initiative. Additionally, the rating scale used to collect evaluation data was biased because it only allowed positive ratings. The open-ended nature of written comments may have provided students with opportunity to make negative statements about the initiative, however, none chose to do so. Despite these limitations, educators in health professional programmes involving dentistry, oral health, and medical laboratory science may find the features of this initiative useful for initiating discussions with colleagues in their institutions.

Given the acceptability of this initiative with facilitators and students, the next step is to consider the feasibility of scaling-up this small voluntary project into a permanent component of the dentistry, oral health, and medical laboratory science programmes. Considerations will include staffing, scheduling, assessment, and attention to cultural perspectives.

## Take Home Messages

• This orofacial pathology IPE initiative addresses a need to promote collaboration and critical assessment skills of oral health information as a team.
• The initiative was based on addressing the competencies of patient-centred care, role clarification, team functioning, collaborative leadership, communication effectively, and cultural practice.
• This reflection-on-practice and description of this initiative may be of interest to educators in health professional programmes involving dentistry, oral health, and medical laboratory science.
• The next version of this initiative must include enhanced learning opportunities that address the cultural practice competency.

## Notes On Contributors

Mrs Hanna Olson, Degree in Dental Hygiene (Gothenburg), MHSc (Kristianstad), Lecturer, Department of Oral Sciences, Faculty of Dentistry, University of Otago, Dunedin, New Zealand. ORCID ID: https://orcid.org/0000-0003-2936-6463

Ms Catherine Ronayne, BMLSc DipGrad (Otago), MNZIMLS, Senior Teaching Fellow, Department of Pathology, University of Otago, Dunedin, New Zealand.

Dr Megan Anakin, BSc (McGill), BFA (NSCAD), BEd (Dalhousie), MEd (Simon Fraser), PhD (Otago), Lecturer and Education Adviser, Education Unit, Dunedin School of Medicine, University of Otago, Dunedin, New Zealand. ORCID ID: https://orcid.org/0000-0002-6499-7802

Mrs Alison Meldrum, BDS, MDS (Otago), Senior Lecturer, Department of Oral Sciences, Faculty of Dentistry, University of Otago, Dunedin, New Zealand. ORCID ID: https://orcid.org/0000-0001-8234-3418

Prof Alison Rich, BDS (Otago) MDSc PhD(Melb) FRACDS FFOP(RCPA), Department of Oral Diagnostic and Surgical Sciences, University of Otago, Dunedin, New Zealand. ORCID ID: https://orcid.org/0000-0001-8096-4245

## Appendices

### Appendix 1

#### Case 1: A patient presenting with oral manifestations of acute leukaemia

*Patient Details:* A 59-year old female patient has made a self-referral to see her oral health therapist. She usually comes for a check-up every 6 months. Although her last visit was only four months ago, she has called in for an emergency appointment because she was worried about her swollen gums.

*Current Concerns:* The patient complains of swollen gums and bleeding from her gums when brushing. Because of this she has not brushed her teeth for a week. General weakness and headache. Loss of appetite.

*General/Physical Appraisal/Social History:* Recent respiratory tract infection, 30 days prior to visit.

*Medical History:* Clear medical history. No allergies.

*Smoking History:* No history of smoking.

*Dental History:* 2-year recall to dentist, attends dental hygienist appointments twice every year.

*Extra-oral examination:* Bruise adjacent to the right lower lip. No recall of specific traumatic injury. Mild enlargement and tenderness of right cervical lymph nodes.

*Intra-oral examination:* Generalized gingival enlargement (Note: Figure 1 is omitted due to copy right). Discrete, bulbous enlargement particularly involving the interdental papillae. Gingival consistency is soft and generally friable. Bleeding from her gums with minor trauma (e.g., toothbrushing), confirmed with periodontal probe. White material on gums can be easily removed with a dental instrument.

The Dentist orders blood tests.

• What key information should be included on the request form? Why are these important (full name, NHI, DOB, requestor, time of collection, non/fasting etc)?
• Why are the tubes different colours and why is this important?
• What information needs to be included on each tube?

What are the consequences of an incorrectly or incompletely labelled tube or request form?

##### Lab results

**Table:**

Full blood count	Patient	Reference interval
Haemoglobin (Hb)	90	135-180 g/L
Mean cell volume (MCV)	87	80-98 fL
Total WBC	35.4	4.0-11.0 x10 ^9^/L
Blasts	30.5	0
Neutrophils	1.0	2.0-7.5 x10 ^9^/L
Lymphocytes	3.4	1.5-3.5 x10 ^9^/L
Monocytes	0.5	0.2-1.0 x10 ^9^/L
Platelets	20	150-450 x10 ^9^/L
Blood film comment(refer to Figures 2A & 2B)	86% blast cells present. Anaemia and marked thrombocytopenia is present. Blood film appearances consistent with acute leukaemia.	
**Chemical pathology**		
Creatinine	125	50-115 umol/L
Uric acid	600	150-470 umol/L
Lactate dehydrogenase (LDH)	1000	230-450 IU/L
Note: Figure 2A is omitted due to copy right.	Note: Figure 2B is omitted due to copy right.	

*Discussion points for the IPE Group*What referrals and investigations may be appropriate now?

• Describe the reason(s) the patient has come to visit her oral health therapist.
• Discuss the risk factors/contributing factors (including systemic factors) involved from an oral health and general health perspective.
• What is the primary role of the dental hygienist when seeing this patient? With whom will they liaise? What would be the pathway of referrals?
• What is the primary role of the dentist when seeing this patient? With whom will they liaise? What would be the pathway of referrals?
• What is the primary role of the medical laboratory scientist involved in the care of this patient? Who can they give the results to?
• Discuss the responsibility of each individual health science profession and how you could work together in managing this case.

#### Case 2: A patient presenting with drug-associated gingival bleeding

*Patient Details:* A 13-year old Caucasian female attended her dentist with her mother because of painful cracks at the corner of her mouth, mouth ulcers and a general feeling of swollen lips. This has been coming and going for over six months.

*General/Physical Appraisal/Social History:* Weight loss of 4kg within the last month and loose stools preceding the oral lesions.

*Medical History:* Jaundice at birth and numerous allergies. No history of medication. No evidence of skin or genital lesions or previous oral lesions.

*Extra-oral examination:* General pallor of the face. No cervical lymphadenopathy. Upper and lower lips swollen, but soft and supple on palpation with surface flaking. Bilateral cracks at corner of mouth (Figure 1A).

*Intra-oral examination:* Folds of hyperplastic tissue bilaterally in the buccal sulcus, with surface ulceration (Figure 1B). Generalised gingival erythema, tongue pale with patchy depapillation.

Clinical Differential Diagnosis:

##### Lab results

**Table:**

Full blood count	Patient	Reference interval
Haemoglobin (Hb)	102	135-180 g/L
Mean cell volume (MCV)*	86	80-98 fL
Mean cell haematology (MCH)	23	26-32 pg
Red cell distribution width (RDW-CV)	22	11.5-14.5%
Total WBC	4.9	4.0-11.0 x10 ^9^/L
Neutrophils	2.2	2.0-7.5 x10 ^9^/L
Lymphocytes	2.3	1.5-3.5 x10 ^9^/L
Monocytes	0.4	0.2-1.0 x10 ^9^/L
Platelets	182	150-450 x10 ^9^/L
Blood film comment(Figure 2A & 2B)	Anaemia is present. Blood film shows hypersegmented neutrophils and red cell anisocytosis with occasional target, pencil, oval and teardrop cells.	
Note: Figure 2A is omitted due to copy right.	Note: Figure 2B is omitted due to copy right.	
**Chemical pathology**
Ferritin	12	20-200 ug/L
CRP	15	<5 mg/L
Vitamin B12	60	211-911 pg/mL
Fecal calprotectin	64	<50 ug/g

*Biopsy of hyperplastic tissue:* Incisional biopsy in relation to right and left buccal mucosa revealed oedematous superficial lamina propria with dilated lymphatic vessels. Lymphocytes were scattered diffusely and in clusters along with fibrous scattered aggregates of non-caseating granuloma. Hematoxylin and eosin staining showed a parakeratinized stratified squamous epithelium along with the underlying granuloma formation under scanner view (Figure 3a) and low power view (3b and c). High power view shows scattered aggregates of non-caseating granuloma (3d and e), which are typically small consisting of macrophages, epithelioid cells surrounded by scattered lymphocytes and plasma cells (3f ) suggestive of the granulomatous lesion. (Note: Figure 3a-f are omitted due to copy right.)

##### Discussion points for the IPE Group

• Describe the reason(s) the patient came to visit the dentist?
• Discuss the differential clinical diagnosis
• What blood tests should be ordered-describe how these tests are ordered, how the blood is obtained and handled at the collection centre and at the diagnostic laboratory
• Why would smears be taken as part of the diagnostic work-up? What are smears, how are they taken and processed?

• Discuss the handling of the biopsy specimen from when it is removed from the lesion until it is given to the pathologist to report on a slide.
• What is the primary role of the dentist when seeing this patient? With whom will they liaise? What would be the pathway of referrals?

• Discuss the responsibility of each individual health science profession and how you could work together in managing this case.
• Discuss other concerns or topics relevant to the case (i.e. complications, systemic involvement, ongoing management).

#### Case 3: A patient presenting with an ulcer subsequently diagnosed as oral cancer

*Patient Details:* A 46-year old woman is visiting her dentist for assessment of a tongue lesion that is non-resolving. She said it is painful when moving the tongue or while eating.

*General/Physical Appraisal/Social History:* No person or family history of recurrent oral ulceration

*Medical History:* Survival after acute myeloid leukemia before age 2. Incident of a cerebral venous sinus thrombosis 8 years ago. Suffers currently from epilepsy, without symptoms for a long time. Takes no medication. No known allergies. Does not drink alcohol. Has no weight changes or night sweats.

*Smoking History:* Has been smoking 2-3 cigarettes/day for 8 years.

*Dental History:* She visits the dentist sporadically, and only when she is in pain.

*Extra-oral examination:* Symmetric face and normal skin colour, motor and sensory cranial nerve functions within normal range. No palpable lymph nodes of either side of the neck. No limitation of the mouth opening.

*Intra-oral examination:* 1.5cm lesion on the right lateral margin of tongue, at about the level of the premolars. It is white and red with a somewhat granular surface with focal ulceration. It is indurated to palpation (Note: Figure 1 is omitted due to copy right).

*Clinical differential diagnosis:*

Traumatic ulcer

Major aphthous ulcer

Ora squamous cell carcinoma (OSCC)

Plan: Need to make a diagnosis, so biopsy or refer

##### Lab results

**Table:**

Full blood count	Patient	Reference interval
Haemoglobin (Hb)	101	135-180 g/L
Mean cell volume (MCV)*	77	80-98 fL
Mean cell haematology (MCH)	23	26-32 pg
Total WBC	10.4	4.0-11.0 x10 ^9^/L
Neutrophils	6.7	2.0-7.5 x10 ^9^/L
Lymphocytes	2.8	1.5-3.5 x10 ^9^/L
Monocytes	0.9	0.2-1.0 x10 ^9^/L
Platelets	245	150-450 x10 ^9^/L
Blood film comment (refer to figures 2A & 2B)	Anaemia is present. Blood film shows pencil cells and target cells.	
Note: Figure 2A is omitted due to copy right.	Note: Figure 2B is omitted due to copy right.	
**Chemical pathology**		
Ferritin	12	20-200 ug/L

*Biopsy of affected tissue:* Nests and islands of squamous cells invading the underlying connective tissue. In some of these tumor islands, there is aberrant keratinization, forming whorls of keratin within (Figure 3A). The invading cells show nuclear pleomorphism and hyperchromatism with brisk mitotic activity (Figure 3B). There is no evidence of neural or vascular invasion. The tumour extends to the deep margin and measures 2.3mm from the surface of the specimen.

**Table:**

Note: Figure 3A is omitted due to copy right.	Note: Figure 3B is omitted due to copy right.

*DIAGNOSIS:* Squamous cell carcinoma, well differentiated, extending to deep margin

Discussion points for the IPE Group

• Describe the reason(s) why the patient has come to visit the dentist?
• Discuss the risk factors/contributing factors (including systemic factors) involved from an oral health and general health perspective.
• Discuss possible differential diagnoses and how to distinguish between them
• What is the primary role of the dentist when seeing this patient? With whom will they liaise? What would be the pathway of referral?
• Discuss the handling of the biopsy specimen from when it is removed from the lesion until it is given to the pathologist to report on a slide.
• Discuss the responsibility of each individual health science profession and how you could work together in managing this case.
• The usual treatment for oral cancer is surgery and or radiotherapy. Discuss the potential complications of these therapies, particularly in light of the age of the patient.
