# Supplementary material for: Working together in clinical pathology: An interprofessional education initiative for dentistry, oral health, and medical laboratory science teachers and students
Source: MedEdPublish (2016). 2020 Aug 28;9:9. Originally published 2020 Jan 13. [Version 2] doi: 10.15694/mep.2020.000009.2 (PMC10697451; doi:10.15694/mep.2020.000009.2)
Supplement: Peer review history

# Reviewer report: Reviewer response for version 2

**DOI:** 10.21956/mep.20263.r31447

**Published:** 2020-09-11

**Authors:** Ken Masters

**Affiliations:** 1 Sultan Qaboos University

This review has been migrated. The reviewer awarded 4 stars out of 5 In this version, I am pleased to see that the authors have included the results of the student evaluation, which have given a far more complete picture of the overall value of the intervention. The gender imbalance of the students could be an issue, but, as the investigation was meant to be a small introduction to investigate the feasibility of upscaling within the institution, it should not be enough to impact negatively on the overall implementation.After implementation, I look forward to seeing a full research paper covering the implementation, with a more detailed evaluation.

Reviewer Expertise:

NA

No decision status is available

# Reviewer report: Reviewer response for version 2

**DOI:** 10.21956/mep.20263.r31446

**Published:** 2020-08-28

**Authors:** Barbara Jennings

**Affiliations:** 1 Norwich Medical School

This review has been migrated. The reviewer awarded 4 stars out of 5 I noted the strengths of the first version of this article, which included 1. sharing the use of authentic case scenarios about oral pathology and 2. the inclusion of useful background literature about the multi-professional approach to managing multiple pathologies 3. the thorough description of this new IPE curriculum. Thank you for this updated version with a more detailed description of how student evaluations were handled by the research team and the description of the research governance steps. The evaluation has highlighted cultural practice as an area for curricular development. It is interesting (and no doubt frustrating to the authors) that a lower SET score for that theme was not accompanied by qualitative comments to follow up on. I enjoyed reading the article and will recommend it to teachers of clinical sciences and those involved with IPE.

Reviewer Expertise:

NA

No decision status is available

# Reviewer report: Reviewer response for version 1

**DOI:** 10.21956/mep.19132.r27631

**Published:** 2020-05-06

**Authors:** Ken Masters

**Affiliations:** 1 Sultan Qaboos University

This review has been migrated. The reviewer awarded 2 stars out of 5 The paper deals with an interprofessional education initiative for dentistry, oral health, and medical laboratory science teachers and students. Frequently, IPE is lauded, but is also very difficult to implement successfully. From the description of the process, the researchers appear to have taken great care to cover the most important information and procedures.A major weakness of the paper, unfortunately, is the lack of evaluation. Although a “New education method or tool” does not require the same rigour as a full research paper, some evaluation is required. The authors have conducted a survey of the students, but have failed to report on the results; instead, they have reported only on the designers’ feelings.If the authors had not conducted a student survey, but had conducted an independent evaluation, that would probably still have been acceptable. Without any results of an evaluation, however, a reader takes away the following message: “They did this thing, and they feel that they did a good thing, but nobody else has said so.” While it is good that the authors feel they have done something valuable, those feelings do not appear to have strong substantiation (or, rather, the substantiation has not been provided).The fact that the authors have conducted a student survey, but have intentionally not reported on the Results (apart from a vague description in the “Outcomes”), however, is deeply troubling:• At worst, they will be accused of intentionally withholding information that could have a material impact on the interpretation of the intervention’s success.• At best, they will be accused of thin-slicing (or salami-slicing) their work in order to get extra publications.Neither of these situations is desirable.Because the journal allows for a Version 2 of a paper, I would strongly recommend that the authors create a Version 2, in which they publish either:• an independent review of their system, and/or• the results of the survey so that readers can have a more accurate picture of the intervention’s value.So, I look forward to seeing Version 2, and to increasing the rating.

Reviewer Expertise:

NA

No decision status is available

# Reviewer report: Reviewer response for version 1

**DOI:** 10.21956/mep.19132.r27630

**Published:** 2020-02-28

**Authors:** Barbara Jennings

**Affiliations:** 1 Norwich Medical School

This review has been migrated. The reviewer awarded 3 stars out of 5 I enjoyed reading this article about cross-faculty curriculum development, and will recommend it to medical educationalists who are interested in the principles and practice of interprofessional education (IPE).In their introduction, the authors present the rationale for this particular application of IPE to undergraduate dentistry and laboratory science students. They explain the value of IPE in general, and the need to increase its reach beyond medics and allied healthcare professionals in practice. The significance of using authentic case scenarios about oral pathology was stressed and the authors cite some useful literature about the multi-professional approach to managing multiple pathologies (though a more recent systematic review about IPE would have been helpful to interested readers). The description of this new IPE curriculum was a key strength for the article. The appendix contains three case scenarios that the sessions centred on. They explore professional relationships but also include useful operational questions, e.g. why are the tubes (for blood tests) different colours, and why is this important? This description about a local educational innovation was considered to be exempt from local ethics approval review processes (because anonymous data was passed from a quality assurance unit to the curriculum designers and authors). But, I wondered if the lack of formal ethical review may have limited the scope of this article and the full description of the evaluation? The evaluation and outcomes section of the article raised unanswered questions for me; e.g. how exactly did the team review and summarise the evaluation data that they collected and reach their conclusions? Also, what about the reflection-on-practice by the facilitators – how was this information captured & evaluated? If any new aspect of a curriculum is likely to be documented in a publication, early and full local ethics board review (of paperwork and processes) is advisable for the sake of the participants and the researchers.

Reviewer Expertise:

NA

No decision status is available
